# Automated Grapevine Cultivar Identification via Leaf Imaging and Deep Convolutional Neural Networks: A Proof-of-Concept Study Employing Primary Iranian Varieties

**DOI:** 10.3390/plants10081628

**Published:** 2021-08-08

**Authors:** Amin Nasiri, Amin Taheri-Garavand, Dimitrios Fanourakis, Yu-Dong Zhang, Nikolaos Nikoloudakis

**Affiliations:** 1 Department of Biosystems Engineering and Soil Science, University of Tennessee, Knoxville, TN 37996, USA; anasiri@utk.edu; 2 Mechanical Engineering of Biosystems Department, Lorestan University, Khorramabad P.O. Box 465, Iran; 3 Laboratory of Quality and Safety of Agricultural Products, Landscape and Environment, Department of Agriculture, School of Agricultural Sciences, Hellenic Mediterranean University, Estavromenos, 71004 Heraklion, Greece; dimitrios.fanourakis82@gmail.com; 4 School of Informatics, University of Leicester, Leicester LE1 7RH, UK; yudong.zhang@le.ac.uk; 5 Department of Agricultural Sciences, Biotechnology and Food Science, Cyprus University of Technology, Limassol CY-3603, Cyprus; n.nikoloudakis@cut.ac.cy

**Keywords:** convolutional neural network, image classification, ImageNet, VGGNet, VGG16, *Vitis vinifera*

## Abstract

Extending over millennia, grapevine cultivation encompasses several thousand cultivars. Cultivar (cultivated variety) identification is traditionally dealt by ampelography, requiring repeated observations by experts along the growth cycle of fruiting plants. For on-time evaluations, molecular genetics have been successfully performed, though in many instances, they are limited by the lack of referable data or the cost element. This paper presents a convolutional neural network (CNN) framework for automatic identification of grapevine cultivar by using leaf images in the visible spectrum (400–700 nm). The VGG16 architecture was modified by a global average pooling layer, dense layers, a batch normalization layer, and a dropout layer. Distinguishing the intricate visual features of diverse grapevine varieties, and recognizing them according to these features was conceivable by the obtained model. A five-fold cross-validation was performed to evaluate the uncertainty and predictive efficiency of the CNN model. The modified deep learning model was able to recognize different grapevine varieties with an average classification accuracy of over 99%. The obtained model offers a rapid, low-cost and high-throughput grapevine cultivar identification. The ambition of the obtained tool is not to substitute but complement ampelography and quantitative genetics, and in this way, assist cultivar identification services.

**Highlights:**

∘ A CNN approach for automatic identification of grapevine cultivar by leaf images.
∘ The VGG16 architecture was modified by adding four layers.
∘ An average classification accuracy of over 99% was obtained.
∘ Rapid, low-cost and high-throughput grapevine cultivar identification was feasible.
○ The obtained tool complements existing methods, assisting cultivar identification services.

## 1. Introduction

In a global perspective, grapevine is one of the most important fruit crops. For *Vitis vinifera* L., the Vitis International Variety Catalogue (VIVC) includes 12,250 variety names, with the actual variety number to be estimated nearly half than that owing to excessive instances of synonyms and homonyms [1]. Ampelography is the science (also referred as art) of variety or cultivar (cultivated variety) identification and classification based on leaf, shoot, and fruit morphological characteristics. Exercising ampelography proficiently requires years of study and practice. The International Office of the Vine and Wine (OIV), the International Union for the Protection of New Varieties of Plants (UPOV) and the International Plant Genetic Resources Institute (IPGRI) have jointly provided the necessary morphological characteristics (Descriptors for Grapevine) for variety identification. Notably, the number of characteristics varies depending on the organization (OIV, UPOV or IPGRI) and the intended use (e.g., gene bank, protection, or systematic classification); still dozens of complex traits across different tissues and epochs must be collected in order to drive meaningful conclusions. Particularly, to achieve the generation of sufficient descriptors, the plant must be grown till the fruiting stage. These characteristics are not simultaneously available at a single phenological stage, necessitating repeated observations along the growth cycle. Trait stability is also of the essence since many of the studied characteristics are not always uniform due to edaphoclimatic discrepancies across regions. Such instances can hamper the successful characterization of cultivars reflecting a lower rate of cultivar registration in plant variety catalogues [2]. In this perspective, the traditional phenotypic approach might be less suitable in instances where a rapid identification is required, or large collections ought to be surveyed.

To counterbalance problematic morphological classification, molecular markers as an alternative, rapid and highly reliable approach for variety identification has gained popularity and is now widely adopted. Several techniques in molecular genetics have been employed in identifying *V. vinifera* (*V. vinifera* subsp. *vinifera* and *V. vinifera* subsp. *sylvestris*) varieties with a high degree of success. Genotyping mainly via the exploitation of simple sequence repeats (SSRs) has been extensively used, as the marker of choice, in order to discriminate accessions, synonyms, parentages and molecular profile delineation [3]. Nonetheless, for routine analysis of cultivar identification, a major limitation of many molecular markers is the lack of universal referable data and harmonization of data (allele sizes) across different laboratories and studies [4]. In many countries, especially in the developing world, the adoption of these approaches is also hampered by the high purchase, maintenance, and operating costs, as well as the need for training personnel to use genetic analyzers and perform sophisticated data analyses.

Instead, imaging in the visible portion of the electromagnetic spectrum (400–700 nm) is not only attainable at low cost, but also easily operated by non-experts [5,6]. In this basis, visible imaging has been increasingly adopted in phenotyping community, including breeders, agricultural industry, and academia [7]. Unlike other organs, leaves can be sampled during most of the growth cycle, have a relatively conserved pattern, while imaging them is a convenient process given their approximately two-dimensional structure. They are characterized by a range of features (e.g., shape, color, texture, edge and venation patterns specific for each cultivar/species [8], which can be employed for identification and classification. Indeed, plant species characterization has been previously performed by features extracted from leaf images through image processing techniques and machine vision [9].

Indeed, indirect, nondestructive methods using regression models for estimating leaves’ dimensions (width and/or length) have recently resolved agronomic and physiologic dilemmas. Such procedures have increasingly found implementation on leaf analysis across several plants such as cacao [10], chestnut [11], fava bean [12], hazelnut [13], ginger [14], maize [15], onion [16] and pepper [17].

Nonetheless, most of the literature has been focused on plant taxon identification, whereas only a handful of studies has addressed (within-species) cultivar identification. In one example of grapevine cultivar identification, leaf red, green and blue (RGB) images were processed to compute 13 features [18]. An artificial neural network (ANN) employing these components successfully performed grapevine cultivar identification [18]. Similar work has been performed in citrus [19]. Despite their proven efficiency, the above-mentioned protocols are based on manual feature engineering in the classification tasks, requiring distinct sections (i.e., feature extraction, selection, and learning). In this perspective, there is a need for new approaches that can automatically handle these distinct sections in a user-friendly manner.

In recent years, deep learning has been effectively employed in many machine vision applications. It uniquely integrates the learning of extracted features and the classification, which are two critical image processing steps. Since features are automatically extracted from raw images in deep learning, manual (hand-crafted) feature extraction is not required. The convolutional neural network (CNN) is a particular class in deep learning. It has been associated with excellent efficiency in object detection, image segmentation, and pattern recognition. In this perspective, the CNNs have been widely employed in addressing complex systems. For instance, CNNs have been successfully employed on plant taxon identification [9].

CNN has many types of layers, which are specifically designed to extract features from images. The two main types are the convolutional and the fully connected layers. The convolutional layer produces an image from another image as input and extracts the specific template from the initial image. For the fully connected layer, the input and output data are transformed vectors.

The initial training process of CNNs requires a large dataset and extensive computational resources. These limitations are commonly overcome by the concept of transfer learning. The transfer learning concept may be employed on CNNs by using the pre-trained CNNs with the learnt weights to extract features. In this method, a selected pre-trained CNN model is applied to extract features from a new dataset, and then a new classifier is used to categorize the extracted features. This type of model is characterized as frozen, meaning that none of the layers within the model can be trained. Alternatively, the transfer learning concept may be employed on CNNs by fine-tuning of the weights in the pre-trained CNN by using a new dataset. In this method, some top model layers must be trained (thus being trainable, also referred to as unfrozen). The method of employing the transfer learning concept on CNNs is determined by (1) the difference in size and (2) the level of similarity between the original data used for pre-training the model and the new dataset.

Despite the significant contribution of Iranian grapevines to the overall global production (accounting approximately for the 5% of global fresh grape crop production; FAO, 2016), misidentification of varieties hampers the full spectra exploitation of the industry’s capacity. The aim of the current study was to employ CNNs for Iranian grapevine cultivar identification. With the aim of being a rapid, low-cost and high-throughput tool, it relies solely on visible leaf images. The objective of the project is not to substitute but complement ampelographic and molecular genetics approaches. The proposed CNN model combined with the traditional phenotypic approach and quantitative genetics can potentially enhance cultivar identification services, and delineate hybrids. For the first time, CNNs are employed for a very fine-grained real-life classification problem.

## 2. Materials and Methods

### 2.1. Plant Material and Sampling Protocol

The six commercially most important grapevine (*V. vinifera* subsp. *vinifera*) cultivars in Iran (Asgari, Fakhri, Keshmeshi, Mirzaei, Shirazi, and Siyah) were employed for the observations. The cultivar collection has been characterized using ampelographic criteria and refers to registered varieties. Vines employed are clones and part of a comprehensive and ongoing research project coordinated by the Research Institute for Grapes and Raisin (RIGR) of Malayer University (Malayer, Iran; latitude 34°, longitude 48°, altitude 1550 m).

Samples were collected in the morning (08:00–10:00 h; 18–20 °C during harvest) in July 2018. Fully expanded leaves from three-year old plants without obvious symptoms of nutrient deficiency, pathogen infection or insect infestation, were sampled from the fifth node (counting from the apex). To retain turgidity, excised leaves were enclosed in plastic envelopes immediately after excision, which were continuously kept under shade [5]. In all cases, the time between sampling and imaging (described below) did not exceed 30 min. Replicate leaves were randomly collected from three separate plants of similar architecture. In total, fifty leaves were sampled per cultivar.

### 2.2. Image Acquisition

**Figure 1:**
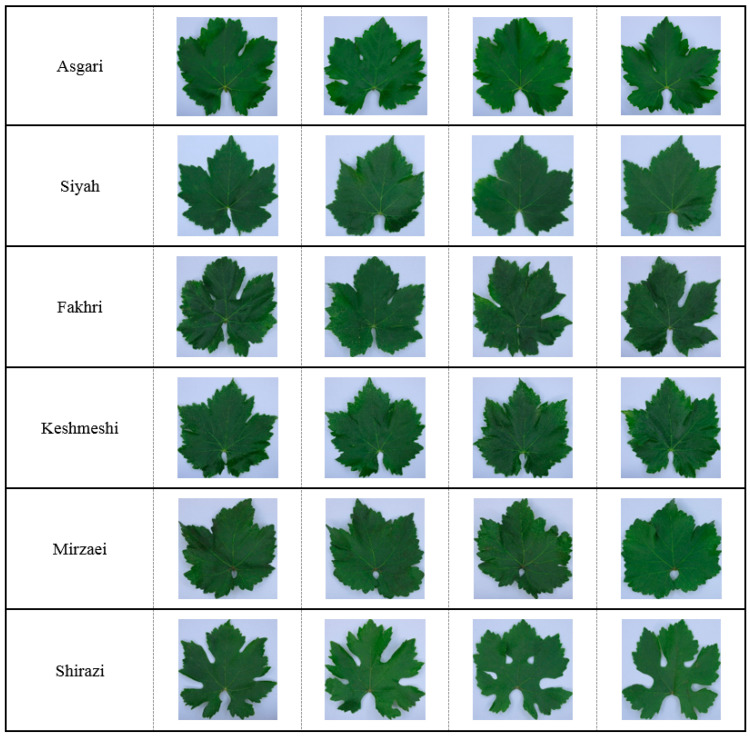
Representative true-to-type leaf images of the six grapevine cultivars under study.

Leaves’ images were obtained by using a charge-coupled device (CCD) digital camera (CANON, SX260 HS, 12.1MP, Made in Japan, specifications in Table S1). The samples were manually moved to the image capture station (Figure S1). The imaging station included four lighting units (halogen tubes; Pars Shahab Lamp Co., Tehran, Iran), located at the corners. The camera-to-sample distance was maintained at 0.3 m. During image acquisition, lighting conditions and image capture settings were fixed. One image (RGB) was obtained per sample. Representative leaf images of different cultivars are shown in Figure 1.

The images were acquired in the RGB color space, and saved in JPEG format as matrices with dimensions of 3000 × 4000 pixels (Table S1). During the training process (described below), the size of the original images was automatically converted to 224 × 224 × 3 (3 = RGB (8 bits each)).

### 2.3. Deep Convolutional Neural Network-Based Model

CNN is an improved version of the traditional ANN. Critical CNN features include local connections, shared weights, pooling process, and the use of multiple layers. In CNN, the learning of abstract features is based on convolutional and pooling layers. Convolutional layer neurons are organized in feature maps. Each neuron is connected to the feature maps of the previous layer through weights matrix (the so-called filter banks). The task of a convolutional layer is to detect local continuities of features from the previous layer. Instead, the task of a pooling layer is to elide similar features into a single feature. Several baseline CNN structures have been developed for image recognition tasks, including Visual Geometry Group network (VGGNet), AlexNet and GoogLeNet.

#### 2.3.1. VGGNet-Based CNN Model

CNN depth affects the classification accuracy. CNN depth is inversely related to classification error. In this perspective, deeper CNN architectures are being explored to improve classification accuracy. To use the advantage of increasing CNN depth, VGGNet proposed a homogeneous convolutional neural network. Although initially intended for object classification tasks, the VGG structure is now widely used as a high-performance CNN. VGGNet has a low structural complexity, while generated features outperform other baseline CNN structures (e.g., AlexNet, GoogLeNet).

VGG16 is one of the two VGGNet structures. It contains 16 weighted layers with 138 million trainable parameters. The homogeneous VGG16 architecture consists of five convolutional blocks. The input of each block is the output of the previous block. Through this structure, VGG16 can extract strong features (e.g., shape, color, and texture) from input images. The first two blocks are constructed by two convolutional layers, while the last three blocks by three convolutional layers. The stride and padding of these 13 convolutional layers with 3 × 3 kernels equal to 1 pixel, while for each of them, the Rectified Linear Unit (ReLU) function is utilized. Every block is followed by a 2 × 2 max-pooling layer with stride 2. This layer is applied as a sub-sampling layer to decrease the feature map dimension. A total of 64 filters are used for the convolutional layers of the first block, and then the number of filters is augmented by a factor of 2 for each consecutive block. The network ends with three dense layers. The first two layers have 4096 channels, and the final layer has a SoftMax activation function with 1000 channels.

The original VGG16 was modified by replacing the last three dense layers with a classifier block. The classifier block included (1) a global average pooling layer, (2) a dense layer with the ReLU function as the activation function, (3) batch normalization to hold the inputs of layers on the same range, (4) dropout as regularization method to reduce the overfitting risk of training, and (5) a final dense layer with SoftMax classifier. The last layer had six neurons to adapt the network to our case study. Various combinations of the dense layers were investigated to obtain the best structure of the classifier block.

**Figure 2:**
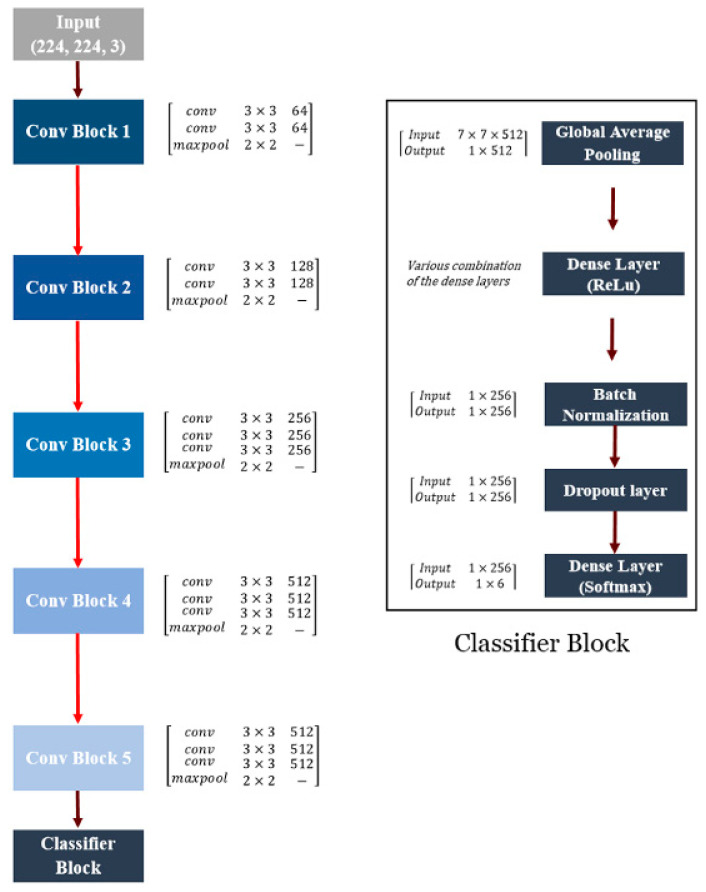
Schematic of VGG-16 architecture (left panel) and classifier block structure (right panel) evaluated in the current research.

Figure 2 denotes the structure of the VGG16-based CNN model. The brackets of convolutional blocks illustrate the type and number of used layers. The second column of the brackets shows the kernel size used in each layer, while the third column represents the number of filters. The brackets of the classifier block indicate the input and output size of layers.

The global average pooling layer minimizes overfitting via reducing the number of parameters. This layer reduces the spatial dimensions of an h × w × d tensor to a tensor with dimensions of 1 × 1 × d. Every h × w feature map is converted to a single number by taking the mean of values of the h × w matrix. The ReLU function performed the mathematical operation (Equation (1)) on every input data, and the SoftMax function calculated the normalized probability value of each neuron ($p_{i}$) by using Equation (2).

(1) $$f\left(x\right)=\{\begin{array}{l} x\;if\;x>0\\ 0\;otherwise \end{array}$$

(2) $$p_{i}=\frac{\exp\left(a_{i}\right)}{\sum_{j=1}^{n}\exp\left(a_{j}\right)}$$

Parameter $a_{i}$ is the softmax input for node *i* (class *i*) and i, j ∈ {1, 2, …, n}.

#### 2.3.2. CNN Model Training

In instances where the training dataset is not extensive, it is not advisable to train the CNN model from scratch. In these instances, it is recommended to utilize a pre-trained CNN model. Fine-tuning was applied for the VGG16-based model through training with the ImageNet dataset. In this way, the knowledge created from ImageNet was transferred to the current case study. Initially, the VGG16 was trained by using the ImageNet. Next, the last fully connected layers were replaced with a new classifier block. This block was trained with random weights for 10 epochs, while all the convolutional blocks were frozen. Finally, the VGG16-based model was trained from scratch with the obtained leaf image dataset. The training process was performed using the cross-entropy loss function and the Adam optimizer with a learning rate and learning rate decay of 1 × 10^−4^ and 2 × 10^−6^, respectively.

**Figure 3:**
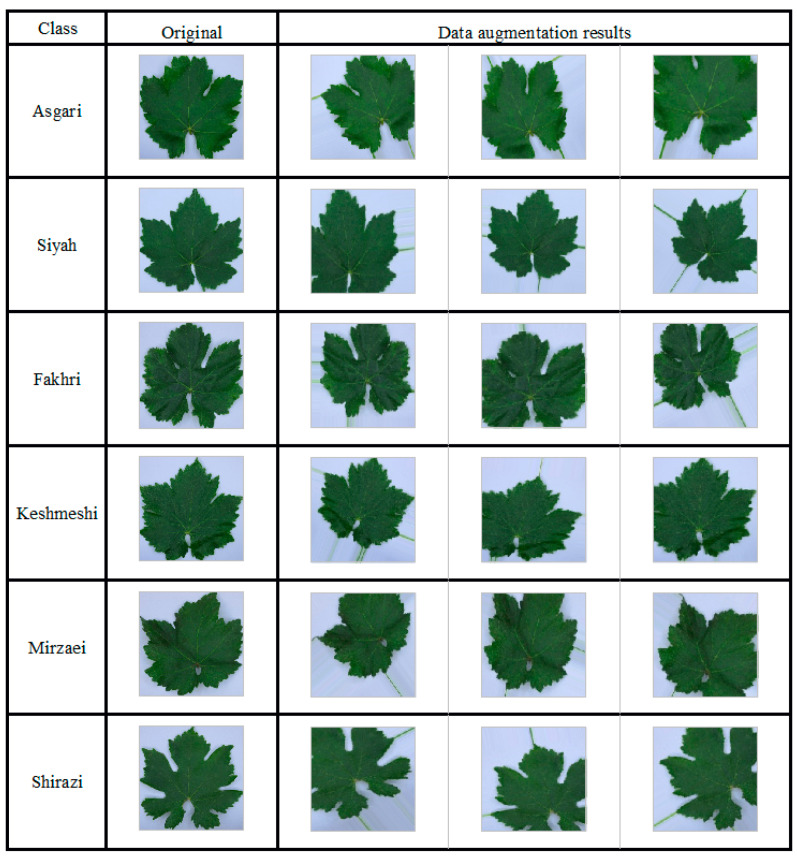
Representative augmented images of the six grapevine cultivars under study.

The VGG16-based model had more than 14 million trainable parameters. Due to the high number of parameters, the overfitting risk of the network would be increased. Owing to the great number of parameters, the network overfitting risk was considered. In order to develop the robustness and generalizability of the network, image processing techniques, a slight distortion (comprised of rotation, height and width shifts), and scaling changes were applied to augment the training of a given image. Representative augmented images of different cultivars are shown in Figure 3.

#### 2.3.3. k-Fold Cross-Validation and Assessment of Classification Accuracy

The image dataset was divided into training and test sets. A five-fold cross-validation was carried out to appraise the predictive performance and uncertainty of the CNN model. To do this, the training dataset was separated into five disjoint equal segments. The CNN model was trained using four (out of five) subsets as the training sets, while the remaining one was used as the validation set. Next, a confusion matrix was calculated to appraise the classification performance on the independent test set. The values of a confusion matrix illustrate the actual and predicted class. This strategy was iterated five times by shifting the validation set. The efficiency of the CNN classifier was expressed by statistical parameters measured based on the values of the confusion matrix in each iteration. These parameters were accuracy, precision, specificity, sensitivity, and area under the curve (AUC). Finally, the average of the five performances was considered as the total performance of the CNN model.

For the training and testing processes, 240 and 60 images were employed, respectively. Based on the number of epochs, k-fold cross-validation method, and the total number of sample batches per training iteration, data augmentation generated 500 new images from each training image. In this way, the training image dataset was 501 times larger than the original one.

## 3. Results and Discussion

*Vitis vinifera* L. (the common grapevine) is widely spread mainly across Eurasia and represents a significant source of revenue to the regional economy [20]. The absence of regulation of reproductive material and the control of grapevine planting encouraged a wide spreading of grapevine types across the years; thus, leading into a surge of novel cultivars [21]. Historically, the certification process is painstakingly conducted by experts using a sophisticated morphological characterization via ampelography [22]. This subject emphasizes on the classification and identification of grapevine cultivars relying on the morphometrics and the features of the grapevine plant; mainly focusing on the form and color of leaves, branches, grape clusters or seeds. Specialists (capable to correctly classify thousands of varieties) are infrequent and the time in order to train professionals is an extremely long investment. To manage this problem, it is vital to technically develop novel and unattended approaches for the correct classification of grapevine varieties, at a quicker frequency, and without elevated expenses when related with ampelography [23].

Nowadays, acquisition of images, image processing, computer visualization and machine learning procedures have been broadly implemented for agricultural purposes. Specifically in the ‘art and science’ of viticulture, significant progress has been made in plant disease classification [24,25,26], nutrient assessment and the berry maturation stage evaluation [27]. Specifically, technical progresses allowed the evaluation of fertilization status of grapevines by using spatial and temporal resolution via unmanned aerial vehicles [28]. Additionally, a two dimensional bud detection scheme by using a CNN for grapevines has been recently established [29]. Grapevine diseases caused by phytoplasmas have also been successfully resolved via CNNs on proximal RGB images [30], while fungal symptoms were earlier detected and successfully classified by using machine learning [31]. Ampatzidis and coworkers [32] also illustrated an innovative grapevine viral disease detection system by combining artificial intelligence and machine learning. Special efforts have focused on applying CNNs and deep learning to the delineation of severe disease such as Esca. Since Esca symptoms can be virtually readily visually evaluated via computer vision and machine learning systems, deep convolutional neural networks can be suitably applied to identify this grapevine disease [33,34,35,36]. Additionally, recognition of grapevine yellow indicators in grapevines via artificial intelligence has been successfully accomplished [37]. Still, such instances are quite straightforward due to the fact that symptomatic leaves develop characteristic alterations in shape (i.e., wilting, leaf rolling) and color (i.e., browning, yellowing) hence, despite challenges, deep CNNs can be readily and successfully applied. Unfortunately, the implementation of deep learning and CNNs is rather limited when it comes to *Vitis* spp. cultivar identification; as a result, reports on this pivotal issue remain infrequent [23].

In the current study, it was attempted to develop a procedure and implement deep learning in a proof-of-concept study in order to efficiently classify the most prominent Iranian grapevine cultivars. In a similar investigation, Fuentes and colleagues successfully classified 16 grapevine cultivars in Spain, via machine learning and near-infrared spectroscopy parameters [18], while Su et al. employed a diverse grapevine dataset for varietal delineation [38]. Recently, real-time on-farm hyperspectral imaging and machine learning were used in order to characterize grapevine varieties using a moving vertical photo system [39]. Such studies are state of the art investigations and have optimal recognition rates. Nonetheless, there is a necessity of expensive equipment (thus not easily attainable across laboratories), while the optimization of sophisticated procedures is required. In order to achieve a robust classification scheme at a fraction of the cost, we selected a common affordable digital camera and an easy to manipulate platform (without sacrificing the classification quality).

The identification of varieties was performed by using a VGG16-based model, which was followed by a classifier block. Several classifier blocks were tested to achieve the best CNN model (Table 1). These were constructed from the global average-pooling layer, by adjusting the number of dense layers. In all cases, these included batch normalization and dropout layers. These networks were trained for 50 epochs in every five folds. The weight matrix of the model was saved based on the lowest loss function value without any over-fitting. The VGG16-based model with three dense layers (including 512, 512, and 256 neurons) provided the best classification outcome and outperformed the others (Table 2). For the training dataset, the average classification accuracy and cross-entropy loss values were 92.72% and 0.2564 (Table 2). For the validation dataset, these parameters were 95.22% and 0.11818, respectively (Table 2). For the validation dataset, these were 97.34% and 0.1203, respectively (Table 2).

**Figure 4:**
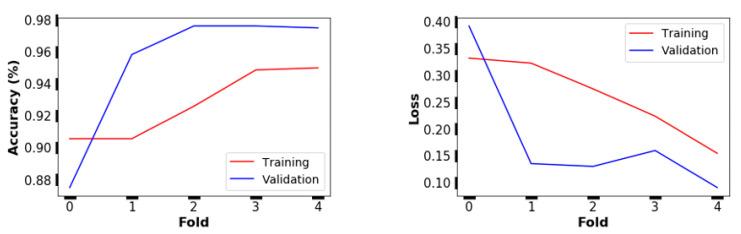
Performance of the VGG16-based model with three dense layers (including 512, 512, and 256 neurons; Table 1) on the training and validation dataset in every five folds.

For that model, the classification accuracy and the cross-entropy loss values of the training and validation dataset are provided in Figure 4. During the training process in each fold, these values were obtained based on the lowest loss function value. The best model efficiency was achieved in the last fold (Figure 4).

**Figure 5:**
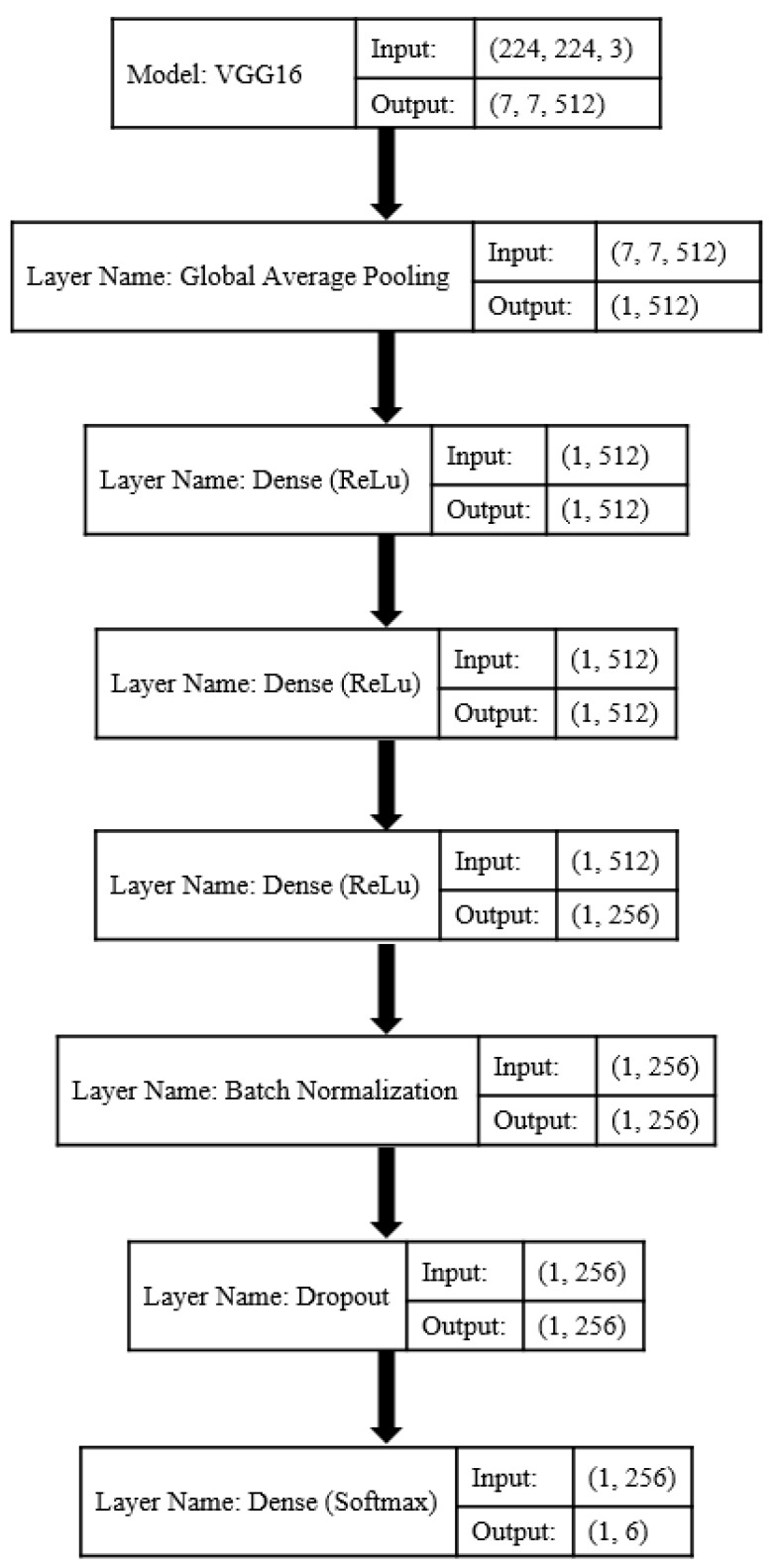
The proposed framework for grapevine cultivar identification.

The proposed framework for grapevine cultivar identification is summarized in Figure 5. The convolutional base of the VGG16 is followed by the developed classifier block. This block contained a global average pooling layer, three dense layers (with 512, 512, and 256 neurons as well as ReLU function), batch normalization layer, dropout layer, and last dense layer with SoftMax classifier. The input and output shape of each layer is also depicted in the figure.

Overall, the recognition model of ANN was successfully applied to varietal leaf classification, and the recognition effect was satisfactory. It was shown that this process can be functional across a wide range of diverse leaf types (shape of blade, number of lobes, shape of teeth petiole sinus etc., as shown in Figure 1), without the utilization of rigorous experimental equipment. In a parallel manner, the model was unattended since it does not need experts’ interference to calibrate the characteristic features, nor did it require background striping of the input images to detect/highlight the main leaf area, more fitting for the scene. Moreover, the training and verification process was self-learning, and highly autonomous having a highly successful recognition rate.

### 3.1. Evaluation of Quantitative Classification

**Figure 6:**
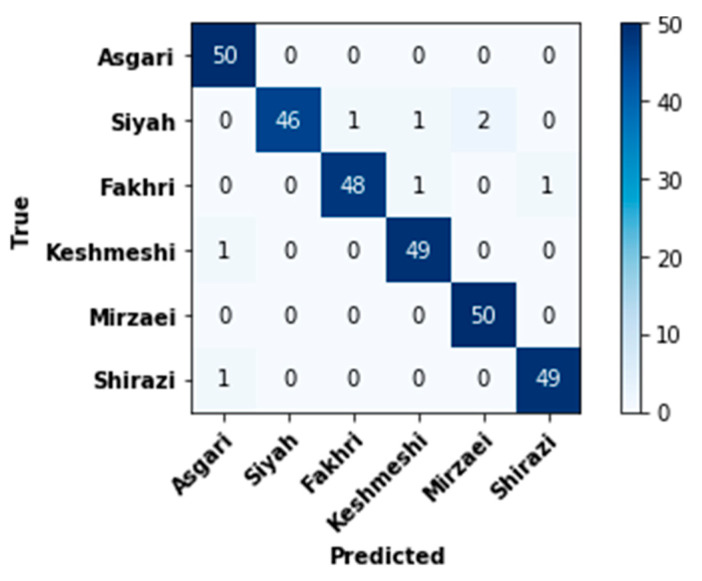
The sum of confusion matrices evaluated from the proposed convolutional neural network model with six classes.

Few samples were misclassified by the selected VGG16-based model (Figure 6). This was noted for one (cvs. Keshmeshi and Shirazi), two (cv. Fakhri) or four (cv. Siyah) predicted samples (Figure 6). Mirzaei and Asgari samples were correctly identified and classified across all tests. This can probably be attributed to discrete features of leaf shapes, since Mirzaei cultivars have small curves and overlaying lobes, while Asgari cultivars discretely display widely angular veins; hence, a clear distinction from other types was established.

In each fold, the performance analysis of the selected VGG16-based model was carried out by the confusion matrix and statistical parameters on the independent test dataset. The total performance of the CNN model was evaluated by calculating the average of all five folds in a range of statistical parameters (accuracy, precision, specificity, sensitivity, and AUC). Finally, the average of the five performances was considered as the total performance of the CNN model (Table 3). The selected VGG16-based model achieved an average overall accuracy of 99.11 ± 0.74% (Table 3). Similarly high values were obtained for the remaining statistical parameters (Table 3).

The prediction efficiency recorded in this study was at the highest end of the range, when considering previous reports assessing cultivar identification by using other methods [18,19]. On top of this, the segmentation, feature extraction, selection, and classification stages were conducted by the model. Instead, in previously published approaches, manual feature engineering is required.

### 3.2. Analysis of Qualitative

The training process of CNN models involves the feed-forward and back-propagation steps. In the back-propagation step, CNN models learn how to optimize their kernels (filters). These optimized kernels extract low- and high-level features and illustrate the most important regions to identify each class (in this case grapevine cultivar). Hence, kernels determine the accuracy of CNN models, and their visualization is needed to evaluate the model performance.

**Figure 7:**
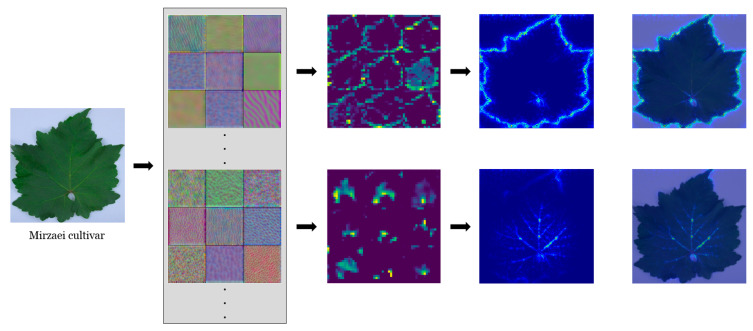
Filter response visualization in a representative leaf of one grapevine cultivar. Convolutional blocks with some filter banks (first dual panel), some channels of the activation layer (second dual panel), and class saliency maps (third and fourth dual panels).

Some kernels (filters) employed in the two convolutional layers (first dual panel) and feature visualization (the so-called feature map; second dual panel) are depicted in Figure 7. The color of specific lamina areas represents some of the learned kernels in the primary layers (first dual panel in Figure 7). These primary layers tended to extract low-level features (e.g., color, edge, polka dot patterns). In the primary layers, the feature map illustrates the active neurons on the leaf blade and edge. The activation of these neurons is possibly associated with the kernels’ focus on color. Notably, the whole leaf boundary is apparent. The neurons activated on the leaf outline are a set of gradient operators, which detected and extracted the leaf silhouette at various orientations. At deeper layers (second dual panel in Figure 7), features at more complex levels were extracted. At these layers, activated neurons included primary and secondary veins.

**Figure 8:**
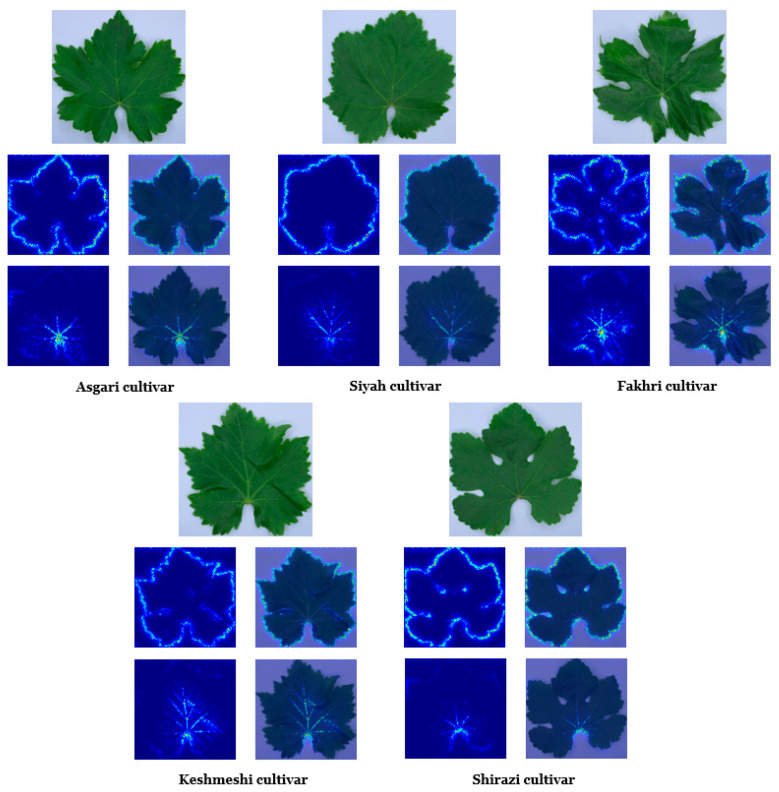
The four picture panel depicts the class saliency map visualization in five grapevine cultivars.

The class saliency map in two convolutional layers is presented (third and fourth dual panels in Figure 7 as well as Figure 8). The class saliency map visualizes the image pixels which contribute to the classification process. For an input image with m rows and n columns, the derivative of class score function was first calculated by back-propagation. The class score function is a linear function of the given image, which can be approximated by the first-order Taylor expansion. Then, the saliency map was computed by rearranging the elements of the calculated derivative. In the RGB image, each component of the saliency map is the maximum value of the computed derivative across the three-color channels.

From both feature (second dual panel in Figure 7) and saliency (third and fourth dual panels in Figure 7 as well as Figure 8) maps, a hierarchy of self-learned features from low to higher levels was observed. Low-level features (e.g., gradient variations along the leaf edge in different directions) were correlated with the higher-level features (e.g., venation-like patterns). This correlation is based on the fact that the higher-level features are built on the low-level ones, and in this way, CNN is capable of constructing robust leaf features. These hierarchical features include color, edge, shape, texture, and venation patterns. Despite the limited cultivar number under study, we were able to show that CNN models are able to realize the complex (visual) leaf features of different grapevine cultivars and, based on these features, distinguish them.

## 4. Conclusions

Thousands of grapevine cultivars are in use today. Their identification is traditionally exercised through the science of ampelography, involving repeated expert observations across the plant growth cycle. On-time grapevine cultivar characterization is currently performed through molecular genetics. However, in several instances, these are limited by the lack of referable data, the involved costs, or the complexed/sophisticated analyses. This study introduced a proof-of-concept convolutional neural network (CNN) framework for automatic identification of grapevine variety by using leaf images in the visible spectrum (400–700 nm). In this perspective, low-cost devices may be employed for unattended image acquisition. The VGG16 architecture was modified by a global average pooling layer, dense layers, a batch normalization layer, and a dropout layer. Distinguishing the intricate visual features of the diverse grapevine varieties, and identifying them based on these features was feasible by the obtained model. A five-fold cross-validation was conducted to assess the uncertainty and predictive efficiency of the CNN model. The obtained model was able to recognize and cluster different grapevine varieties with an average classification accuracy of over 99%. This sets it as a rapid, low-cost and high-throughput technique for grapevine variety identification. The objective of the obtained methodology is not to replace but complement current methods (ampelography and molecular genetics), and, in this way, reinforce cultivar identification services.

## Figures and Tables

**Table 1:** Structure of the classifier blocks tested in this study. Brackets show the number of the dense layers and their neurons.

Model	Layer Name				
	Global Average Pooling	Dense (ReLU)	Batch Normalization	Dropout	Dense (Softmax)
1	✓	[256]	✓	✓	[6]
2	✓	[512]	✓	✓	[6]
3	✓	[512, 512]	✓	✓	[6]
4	✓	[512, 512, 256]	✓	✓	[6]

✓ The used layer in the model construction.

**Table 2:** Comparison of the proposed convolutional neural networks performance in various layer combinations provided from Table 1.

Model	Training				Validation				Test			
	Accuracy		Loss		Accuracy		Loss		Accuracy		Loss	
	Average	STD	Average	STD	Average	STD	Average	STD	Average	STD	Average	STD
1	0.84	0.05	0.49	0.09	0.9	0.05	0.43	0.18	0.93	0.04	0.25	0.08
2	0.87	0.04	0.41	0.08	0.91	0.05	0.33	0.15	0.98	0.02	0.17	0.08
3	0.91	0.03	0.3	0.11	0.93	0.05	0.26	0.17	0.97	0.03	0.12	0.08
4	0.93	0.02	0.26	0.07	0.95	0.04	0.18	0.12	0.97	0.02	0.12	0.06

**Table 3:** Average statistical parameters and deviations of the selected convolutional neural network model. AUC = area under the curve.

Class	Accuracy (%)		Precision (%)		Sensitivity (%)		Specificity (%)		AUC (%)	
	Average	STD	Average	STD	Average	STD	Average	STD	Average	STD
Asgari	99.34	1.49	96.67	7.45	100	0	99.2	1.79	99.6	0.89
Siyah	98.67	2.17	100	0	92	13.03	100	0	96	6.52
Fakhri	99	0.91	98.18	4.06	96	5.47	99.6	0.89	97.8	2.58
Keshmeshi	99	0.91	96.36	4.98	98	4.47	99	1.09	98.6	2.07
Mirzaie	99.34	0.91	96.36	4.98	100	0	99.2	1.09	99.6	0.54
Shirazi	99.34	0.91	98.18	4.06	98	4.47	99.6	0.89	98.8	2.17
Average per class	99.11	0.74	97.62	1.96	97.33	2.23	99.47	0.44	98.4	1.34

## Data Availability

All relevant data are included in the manuscript. Raw images are available on request from the corresponding author.

## Supplementary Materials

The following are available online at https://www.mdpi.com/article/10.3390/plants10081628/s1, Figure S1: The image capture station employed in the current study, Table S1: Camera specifications employed in this study.

## Abbreviations

ANN	artificial neural network
AUC	area under the curve
CCD	charged-coupled device
CNN	convolutional neural network
IPGRI	International Plant Genetic Resources Institute
OIV	International Office of the Vine and Wine
ReLU	Rectified Linear Unit
RGB	red, green and blue
RIGR	Research Institute for Grapes and Raisin
UPOV	International Union for the Protection of New Varieties of Plants
VGGNet	Visual Geometry Group network
VIVC	Vitis International Variety Catalogue
