# Supplementary material for: Automated Grapevine Cultivar Identification via Leaf Imaging and Deep Convolutional Neural Networks: A Proof-of-Concept Study Employing Primary Iranian Varieties
Source: Plants (Basel). 2021 Aug 8;10(8):1628. doi: 10.3390/plants10081628 (PMC8399703; doi:10.3390/plants10081628)
Supplement: Supplementary file 1 [file plants-10-01628-s001.zip › plants-1271057-SI.pdf]

Supplementary Table S1. Camera specifications employed in this study.

Variable	Value/ State
Image size	3000*4000 pixels
Zoom	No zoom
Flash mode	No flash
White balance	florescent
Sensitivity	ISO-200
Aperture Av.	f/4.5
Exposure time Av.	1/20 s
Resolution	180 dpi
Focal length	1/500 mm
Operation mode	Manual
Macro	Off
Image type	JPEG

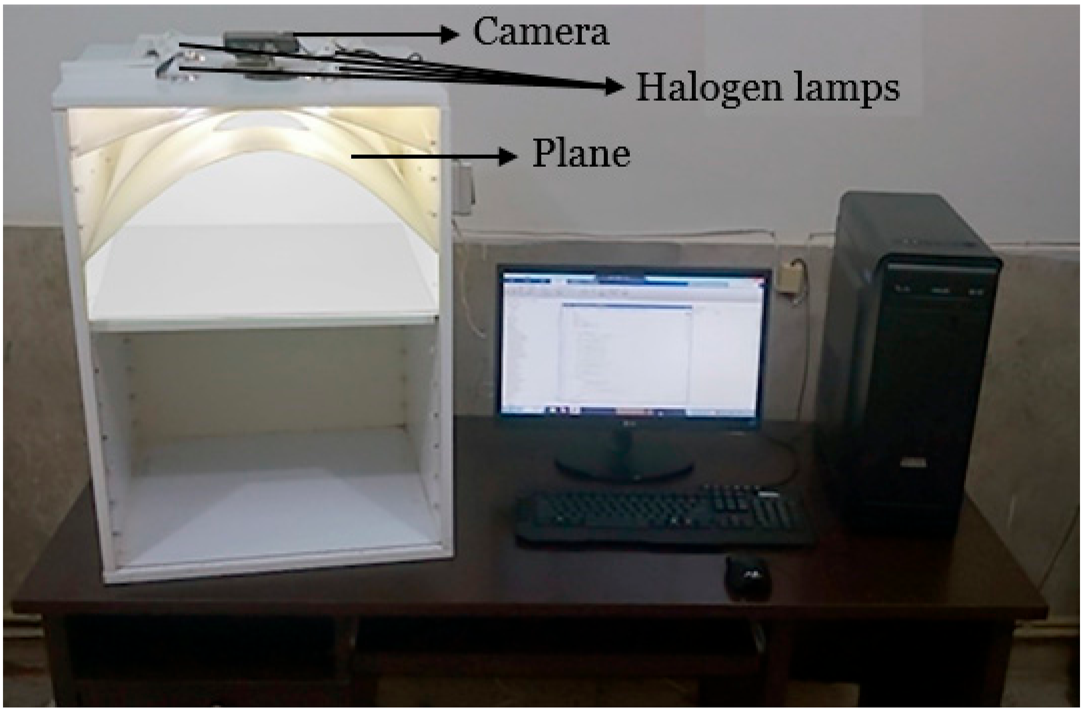

Supplementary Figure S1. The image capture station employed in the current study.
